# Ischemic stroke induces cardiac dysfunction and alters transcriptome profile in mice

**DOI:** 10.1186/s12864-021-07938-y

**Published:** 2021-09-04

**Authors:** Jie Chen, Jiahong Gong, Haili Chen, Xuqing Li, Li Wang, Xiaoli Qian, Kecheng Zhou, Ting Wang, Songhe Jiang, Lei Li, Shengcun Li

**Affiliations:** 1 Rehabilitation Medicine Center, The Second Affiliated Hospital and Yuying Children’s Hospital of Wenzhou Medical University, Wenzhou, 325027 Zhejiang China; 2 Integrative & Optimized Medicine Research center, China-USA Institute for Acupuncture and Rehabilitation, Wenzhou Medical University, Wenzhou, 325027 Zhejiang China; 3 Key Laboratory of Chinese Internal Medicine of Ministry of Education and Beijing, Dongzhimen Hospital, Beijing University of Chinese Medicine, Beijing, China; 4 Institute of Cardiovascular Development and Translational Medicine, The Second Affiliated Hospital and Yuying Children’s Hospital of Wenzhou Medical University, Wenzhou, Zhejiang China

**Keywords:** Ischemic stroke, Cardiac dysfunction, Atrophy, Transcriptome

## Abstract

**Background:**

Stroke can induce cardiac dysfunction in the absence of primary cardiac disease; however, the mechanisms underlying the interaction between the neurological deficits and the heart are poorly understood. The objective of this study was to investigate the effects of stroke on cardiac function and to identify the transcriptome characteristics of the heart.

**Results:**

Stroke significantly decreased heart weight/tibia length ratio and cardiomyocyte cross-sectional areas and increased *atrogin-1* and the E3 ubiquitin ligase *MuRF-1*, indicating myocardial atrophy in MCAO-induced mouse hearts. RNA sequencing of mRNA revealed 383 differentially expressed genes (DEGs) in MCAO myocardium, of which 221 were downregulated and 162 upregulated. Grouping of DEGs based on biological function and quantitative PCR validation indicated that suppressed immune response and collagen synthesis and altered activity of oxidoreductase, peptidase, and endopeptidase may be involved in MCAO-induced cardiomyopathy. The DEGs were mainly distributed in the membrane or extracellular region of cardiomyocytes and acted as potential mediators of stroke-induced cardiac dysregulation involved in cardiac atrophy.

**Conclusion:**

Stroke induced a unique transcriptome response in the myocardium and resulted in immediate cardiac atrophy and dysfunction.

**Supplementary Information:**

The online version contains supplementary material available at 10.1186/s12864-021-07938-y.

## New and noteworthy

Our study revealed three new major findings: (1) experimental induced stroke caused cardiac dysfunction, (2) atrophic phenotype of the heart occurred after induced stroke, and (3) a unique myocardial mRNA expression profile was produced after induced stroke in mice.

## Introduction

Cardiovascular and cerebrovascular diseases remain among the primary causes of death and medical costs worldwide [1], and share several risk factors [2]. Although it is well known that cardiogenic strokes contribute to the occurrence of ischemic stroke [3], increasing evidence also suggest that elevated cardiac troponin [4] and left atrial enlargement [5] occur in patients with stroke. These cardiac biomarkers (cardiac troponin and left atrial diameter) are associated with the presence of large vessel occlusions [6]. Myocardial injury, ischemia-like electrocardiographic changes and arrhythmias, frequently occur in acute stroke patients, even in the absence of primary heart disease [2]. In this study, we define heart disease of central nervous system origin as **stroke-induced cardiomyopathy**. Cardiac dysfunction induced by stroke is discussed in several animal experiments [7, 8]. Focal cerebral ischemia induces long-term cardiac dysfunction, with reduced left ventricular ejection fraction (EF) and increased left ventricular volumes; these are associated with higher peripheral sympathetic activity [9]. In addition to impairing cardiac contractility, stroke also leads to greater myocardial vulnerability [10]. One study reported that cerebral ischemic stroke in adult mice induces chronic cardiac dysfunction, fibrosis, hypertrophy and secondary immune response, and may contribute to post stroke cardiac dysfunction [11]. Conversely, another study indicated that transient cardiac atrophy, and not fibrosis and hypertrophy, is induced, owing to up-regulation of the E3-ligase atrogin-1 and up-regulation of Pparg-dependent genes [12]. The mechanisms of conventional cardiac injury, such as sympathetic regulation [9], inflammation [11], and autophagy [13] are considered to be involved in the process of cardiomyopathy after stroke. Historically, stroke therapeutic strategies have focused on treating two parts separately; targeting the central nervous system for brain injury, and treating systemic complications as their own entity [14]. Although the previous study has found some differential molecular expression in the heart muscle 14 days after stroke [12], the underlying molecular mechanisms of stroke-induced neurogenic cardiac dysfunction are yet to be established and we did research at a different point in time than the previous study.

Herein, we first analyzed cardiac function and myocardial remodeling in an induced stroke mouse model. We then used next generation RNA sequencing (RNA-seq) technology to investigate the transcriptome change in mouse heart after stroke. Finally, we described and analyzed the bioinformatics functions of differentially expressed genes (DEGs) in the heart muscle. Understanding the direct mechanisms of stroke-induced cardiomyopathy adequately will be valuable for the development of targeted therapies and promoting stroke rehabilitation.

## Materials and methods

### Animals and cerebral ischemia model

All experiments were approved by the Institutional Animal Care and Use Committee of Wenzhou Medical University and performed in accordance with the National Institute of Health’s Guide for the Care and Use of Laboratory Animals. Male (8–9 weeks) C57BL/6 mice were purchased from the Model Animal Research Centre of Wenzhou Medical University. Mice were housed at a constant temperature of 22 °C in a 12/12-light/dark cycle with free access to regular rodent chow and tap water. Mice were randomly assigned to two groups according to the random number table (*n* = 6, each group) as follows: Sham group and MCAO group.

Focal cerebral ischemia was induced by transient middle cerebral artery occlusion (MCAO) for 60 min, as described previously [12, 15]. Briefly, mice were anesthetized with 1.5% isoflurane in O_2_. A thermostatic heating blanket was used to maintain core body temperature close to 37 °C during surgery. After a midline neck incision, a standardized silicone rubber–coated No.6.0 nylon monofilament was inserted into the right carotid artery and advanced via the internal carotid artery to occlude the origin of the middle cerebral artery. After 60 min, mice were re-anesthetized, and the occluding filament was removed to allow reperfusion. Mice subjected to MCAO with neurological deficit scores of 2–3 were used in subsequent experiments. Exclusion criteria of MCAO group are as follows: mice with subarachnoid hemorrhage; TTC staining showed no obvious infarct size in brain tissue section; mice died within 24 h after operation. Mice with the conditions above need to be excluded. Owing to the mortality rate of about 30% in MCAO group, the shortfall needs to be made up. We performed these experiments under the ARRIVE (Animal Research: Reporting of In Vivo Experiments) guidelines. Ethical approval for all experimental procedures was granted by the Animal Ethical and Welfare Committee at Wenzhou Medical University.

### Cardiac function measurement

Mice were anesthetized with isoflurane (1%) and an animal ultrasound system Vevo2100 (VisualSonics, Canada) equipped with a 40 MHz pediatric transducer, which was used to record an echocardiogram. M-mode and 2-D parasternal short-axis scans at the level of the papillary muscles were used to assess changes in the left ventricle (LV) end-systolic inner diameter, LV end-diastolic inner diameter, and fractional shortening (FS, %) [16, 17]. To assess diastolic function, we obtained apical four-chamber views of the LV. The pulsed wave Doppler measurements of maximal early (E) and late (A) transmitral velocities in diastole were obtained in the apical view with a cursor at the mitral valve inflow [18].

### Assessment of neurological deficit and cerebral infarction

Neurological deficit was assessed in each animal at 2 h, 24 h and 4 days following MCAO in a blinded manner using a 4-point evaluation as described previously [19]. Mice subjected to MCAO with neurological deficit scores of 2–3 were used in subsequent experiments. After obtaining the heart, quickly remove the brain of mice. The cerebral infarction was identified using 2, 3, 5-triphenyl-tetrazolium (TTC) staining for sections of brains. .

### Histological analyses

Cardiac sections sized 5 μm were cut from paraffin-embedded tissue. H&E staining was used for pathological evaluation of the hearts. The myocardial collagen was assessed by staining with Picrosirius red (Solarbio, China), the area of total collagen deposition was measured by Image-Pro Plus 6.0 software. Cross-sectional areas of cardiomyocytes were measured by ImageJ software after staining for cell membranes and nuclei with fluorescein isothiocyanate–conjugated wheat germ agglutinin (WGA) (Thermo Fisher Scientific, USA) and Hoechst33342 (Thermo Fisher Scientific, USA), respectively.

### RNA-seq data analysis and library construction

Total RNA was extracted using mirVana miRNA Isolation Kit (Shanghai OE-Biotech) following the manufacturer’s protocol [20]. RNA integrity was evaluated using Agilent 2100 Bioanalyzer (Agilent Technologies, Santa Clara, CA, USA). The samples with RNA Integrity Number (RIN) > 7 were subjected to further analysis. The libraries were constructed using TruSeq Stranded mRNA LT Sample Prep Kit (Illumina, San Diego, CA, USA) according to the manufacturer’s protocol. Then, these libraries were sequenced on the Illumina sequencing platform (HiSeqTM 2500 or Illumina HiSeq X Ten) and 125 bp/150 bp paired-end reads were generated.

### Gene ontology (GO) and network analysis

Cufflinks quantitatively evaluated the RPKM (reads per kilobase million) value of each gene [21]. HTSeq-count calculated the read counts of each gene [22]. Differential expression analysis was performed using the DESeq R package [23]. The negative binomial distribution test method (NB) was used to verify the significance of the difference in the number of reads. The *P*-value < 0.05 adjusted by false discovery rate (FDR) and fold change (FC) > 2 or < 0.5 were set as the thresholds for significant differential expression. GO enrichment and KEGG (Kyoto Encyclopedia of Genes and Genomes) pathway enrichment analysis [24] of DEGs were respectively performed using R package, based on the hypergeometric distribution [25]. The number of counts of each sample gene was normalized using DESeq software [6] (basemean value was used to estimate the expression quality), the fold change of difference was calculated, and the number of reads was tested for the significance of difference using NB (negative binomial distribution test). Finally, the differential protein-coding genes were screened based on the fold change of difference and the significance of difference. All protein-coding genes were used as the background list, and the list of differential protein-coding genes was used as the candidate list screened from the background list. The hypergeometric distribution was used to calculate the *P*-value representing whether the GO function was significantly enriched in the list of differential protein-coding genes, and then the *P*-value was corrected by Benjamini & Hochberg multiple test to obtain the FDR. Pathway analysis of differential protein-coding genes was performed using the KEGG database [7], and the significance of DEG enrichment in each pathway entry was calculated using a hypergeometric distribution.

### Quantitative PCR (qPCR)

Total RNA was isolated from the mouse heart tissue using TRIzol reagent (TaKaRa, Dalian, China), and 1000 ng of RNA was reverse-transcribed to cDNA using the first-strand synthesis system for PCR (Invitrogen, Carlsbad, CA, USA) according to the manufacturer’s protocol. The cDNA was used as a template in qPCR to analyze the mRNA expression levels of atrial natriuretic peptide (ANP), beta-myosin heavy chain (β-MHC), collagen-1 and -3, IL-1β, IL-6 and TNF-α genes, along with selected DEGs. The result comparison between sham group and MCAO group was tested by Student’s t-test.

All primer sequences used in this study are listed along with their accession numbers in Supplemental Table S1.

### Western blotting and ELISA

Protein levels were analyzed using Western blotting or ELISA according to the instructions.

### Statistical analysis

All data are presented as mean ± SD. The level of statistical significance was determined using one-way analysis of variance (ANOVA). Student’s t-test was used for comparison between two groups. Statistical analyses were performed using GraphPad Prism v5.0 (GraphPad Software, San Diego, CA, USA). A *P-*value < 0.05 was considered to be statistically significant.

## Results

### MCAO*-*induced cardiac dysfunction

**Fig. 1:**
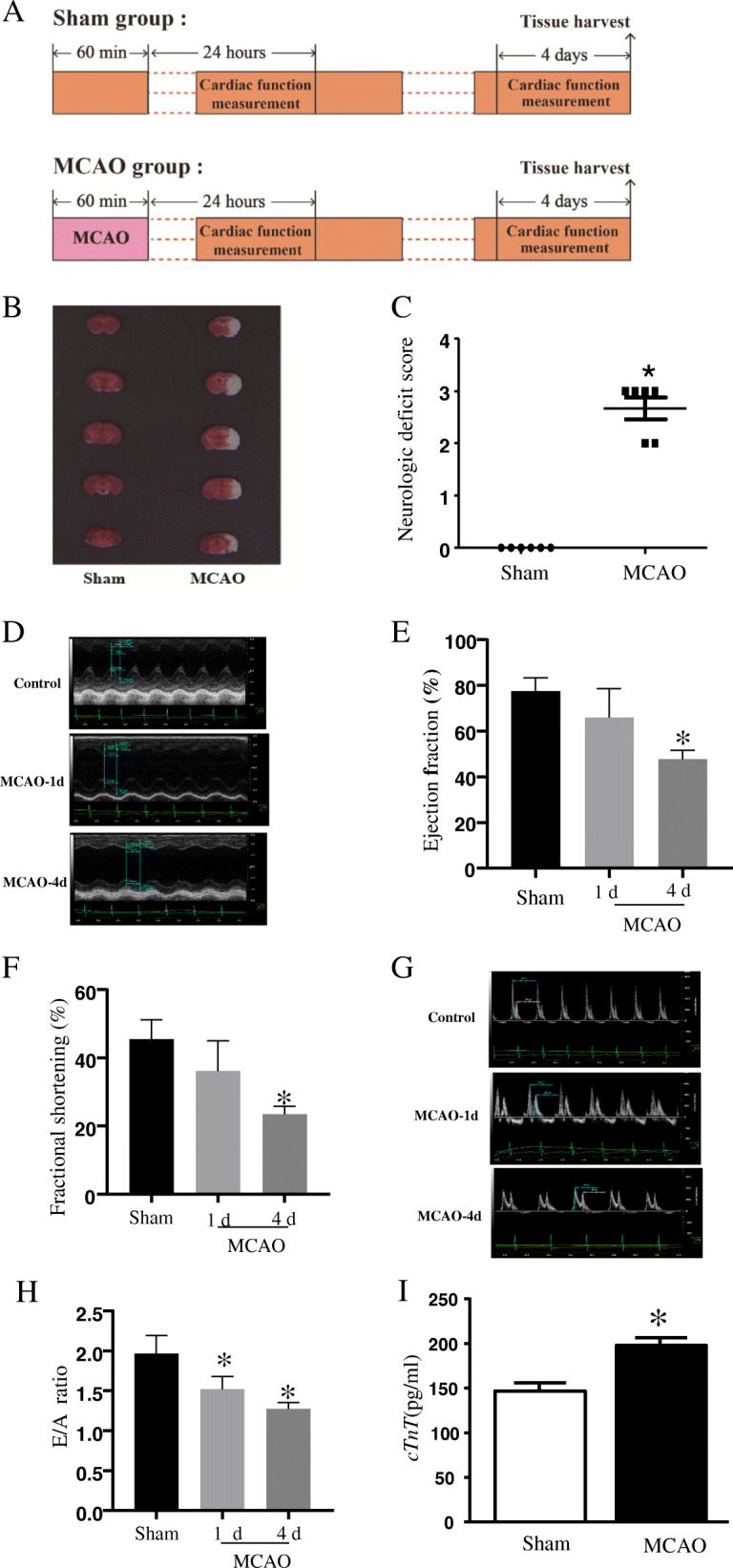
Consequences of stroke on cardiac function. (**A**) Schematic diagram of the mice models, including sham group and MCAO group; (**B**) Right hemispheric brain samples were stained with triphenyl tetrazolium chloride (TTC) 24 h after a 60 min middle cerebral artery occlusion (MCAO). (**C**) Neurological deficits of MCAO-induced mouse. (**D**) Representative view at the papillary muscle level of echocardiography (M-model). (**E**) Ejection fraction (EF, %) and (**F**) fractional shortening (FS, %). (**G**) A representative mitral E/A image on Doppler echocardiography. (**H**) E/A ratio. (**I**) cTnT level in serum. Data are presented as mean ± SD, *n* = 6. *P < 0.05 vs sham-operated

To gain insight into the potential molecular changes in MCAO-induced cardiac dysfunction model, we first established an in vivo mice model of middle cerebral artery occlusion, and performed cardiac ultrasound on the mice on the first and fourth days, as shown in Fig. 1A. Figure 1B and C show cerebral infarction and neurological deficits following induced cerebral ischemia, indicating that the MCAO model was established successfully in this study. Understanding the pathophysiological changes in the early stages of stroke is important for the development of rehabilitation and treatment strategies. Therefore, we focused on the acute changes in cardiac function after stroke within 1–4 days. Cardiac function impairment was detected in the mice 24 h after stroke, which further decreased at 4 days compared with that of the sham-operated mice (Fig. 1D). As shown in Fig. 1E and F, stroke decreased the EF% and FS% as markers of systolic function deterioration. Similarly, progressive impairment of diastolic function was identified by a reduced E/A ratio in MCAO mouse hearts (Fig. 1G and H). In the initial state of anesthesia, the average heart rate of MCAO group decreased compared to sham group (Supplemental Table S2). cTnT is an important marker of myocardial injury. We used ELISA to detect the value of cTnT in each group. The results showed that cTnT was increased in MCAO mouse heart (Fig. 1I).

### MCAO led to cardiac atrophy and myocardial remodeling

**Fig. 2:**
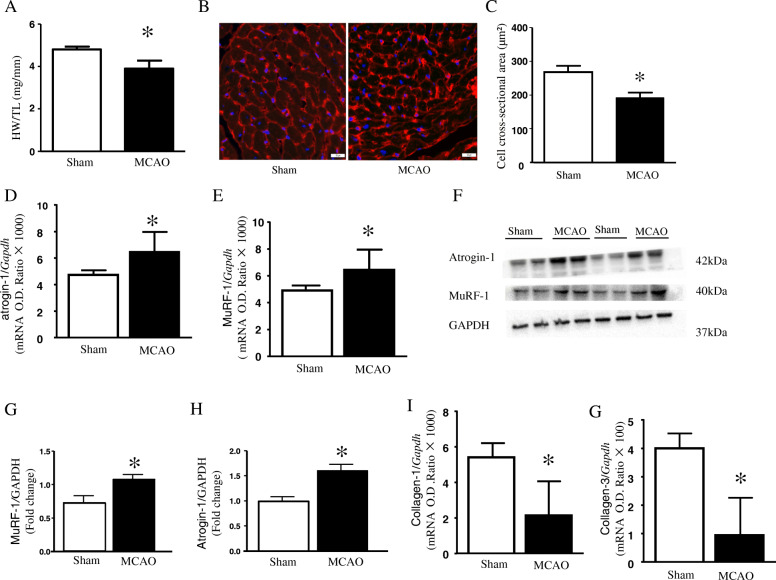
Myocardial remodeling in the heart after stroke. (**A**) Heart weight/tibia length ratio (WT/TL) at 4 days after stroke. (**B**) Representative wheat germ agglutinin (WGA)-Hoechst images in mouse heart and (**C**) Cardiomyocytes cross-sectional areas quantification. (**D-E**) The mRNA levels of atrogin-1 and MuRF-1. (**F-H**) The protein levels of atrogin-1 and MuRF-1. (**I-G**) The mRNA levels of collagen-1, and collagen-3. Data are presented as mean ± SD, *n* = 6. *P < 0.05 vs sham-operated

As dramatic weight loss occurred in mice after stroke, we speculated that myocardial atrophy may occur. Heart weight/tibia length ratio was reduced after stroke, compared with the sham-operated mice (Fig. 2A). Histological analysis of cardiomyocyte cross-sectional areas also revealed that cardiomyocyte size decreased in MCAO mouse hearts (Fig. 2B and C). Hematoxylin and eosin (HE) staining showed that cardiomyocytes shrank, and extracellular matrix cavities became larger in MCAO mouse tissue (Supplemental Fig. S1A). Furthermore, mRNA and protein expression levels of *atrogin-1* (Fig. 2D and F) and *MuRF-1* (Fig. 2F-H) were up-regulated in MCAO mouse hearts (full blots see in Supplemental Fig. S3).

In addition, we detected the expression of marker genes of cardiac hypertrophy and fibrosis.. We found that hypertrophic expression of the β-MHC gene (Supplemental Fig. S1B) was upregulated, as well as the level of ANP mRNA (Supplemental Fig. S1C). Unexpectedly, the mRNA expression levels of collagen-1 (Fig. 2I) and collagen-3 (Fig. 2G), which are classically involved in pathological myocardial remodeling, were reduced in the stroke-induced mouse hearts. However, Sirius red staining did not demonstrate a change in the deposition of total collagen in the MCAO-induced mouse hearts (Supplemental Fig. S1D).

### DEGs in stroke-induced mouse hearts

**Fig. 3:**
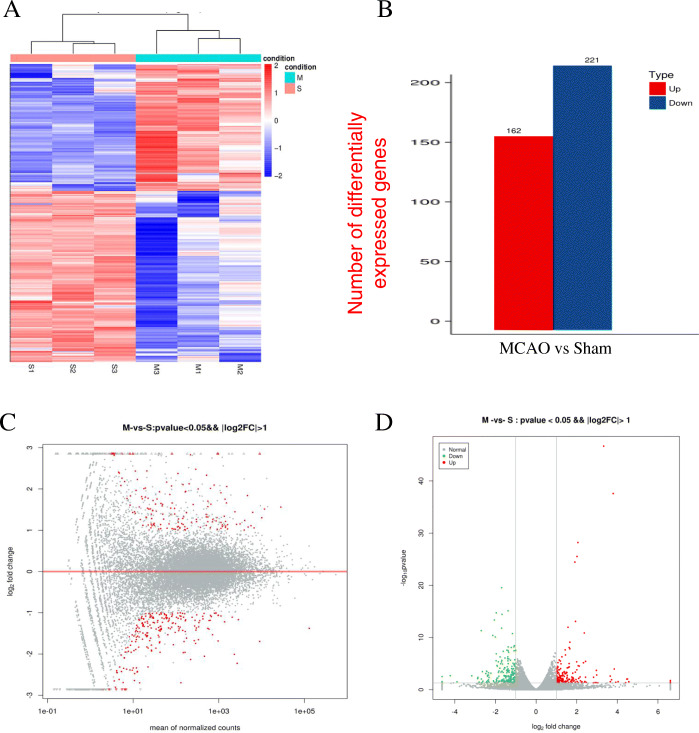
Transcriptome genes expression altered in stroke-induced heart. (**A**) Hierarchal clustering of 383 differentially expressed genes (DEGs) in middle cerebral artery occlusion (MCAO) hearts found using RNA sequencing (RNAseq). (**B**) 221 DEGs were downregulated while 162 were upregulated in stroke mouse hearts. (**C**) DEGs separated (MA plot) in 1-fold change (FC) comparing with sham-operated group. (**D**) A volcano plot of DEGs

High throughput sequencing was performed using cardiac RNA from MCAO mouse hearts after 40 h. First, we obtained DEGs using cutoff FC > 2 and FC > 2 and cutoff FC < 0.5 for both up−/down-regulated genes between MCAO and sham-operated mice (Fig. 3A). Bioinformatics analysis revealed a total of 383 DEGs in the MCAO mouse hearts for which 221 transcripts showed decrease in abundance, while 162 genes showed an increase (Fig. 3B). We listed the top ten differentially upregulated miRNA genes and the downregulated miRNA genes in Table 1 and in Table 2 in Gene ID forms. In order to present the overall distribution of DEGs, we drawn a MA plot (Fig. 3C) and a volcano plot (Fig. 3D) with a threshold of log2FC > 1. In a MA plot, significant DEGs are marked in red. In a volcano plot, significant DEGs were marked in red and green.

**Table 1:**
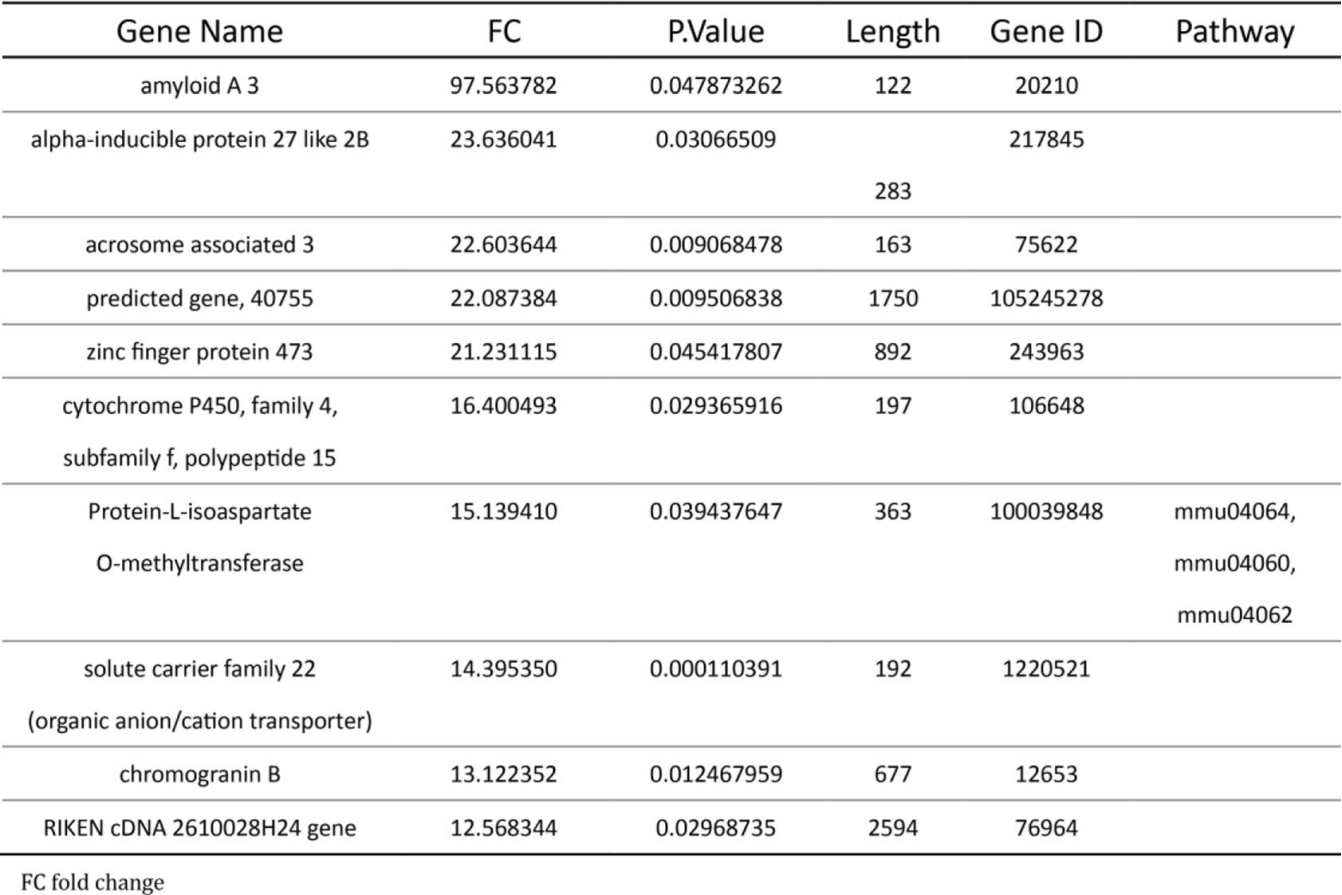
Basic and obstetric characteristics of SSI and control groups

**Table 2:**
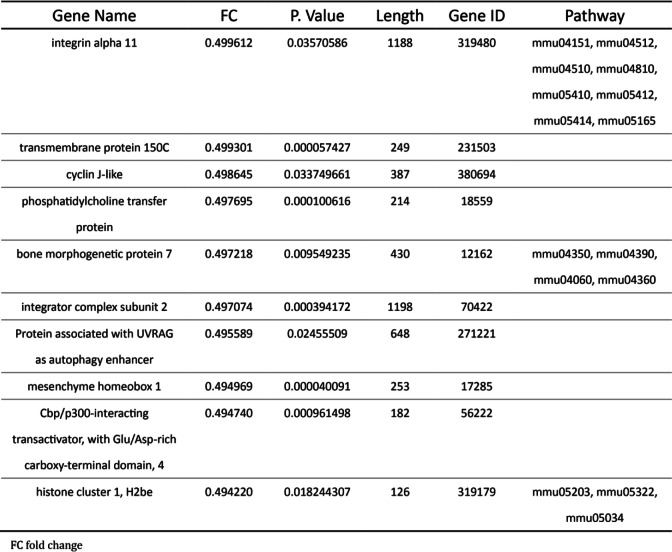
Multivariate analysis of risk factors for SSI

### GO analysis of DEGs in stroke-induced mouse hearts

To further understand the gene changes associated with stroke-induced cardiomyopathy, GO enrichment analysis was performed by clusterProfiler on 2770 GO-terms, with 1405 Down-terms (*P* < 0.05) and 1063 Up-terms (*P* < 0.05).

**Fig. 4:**
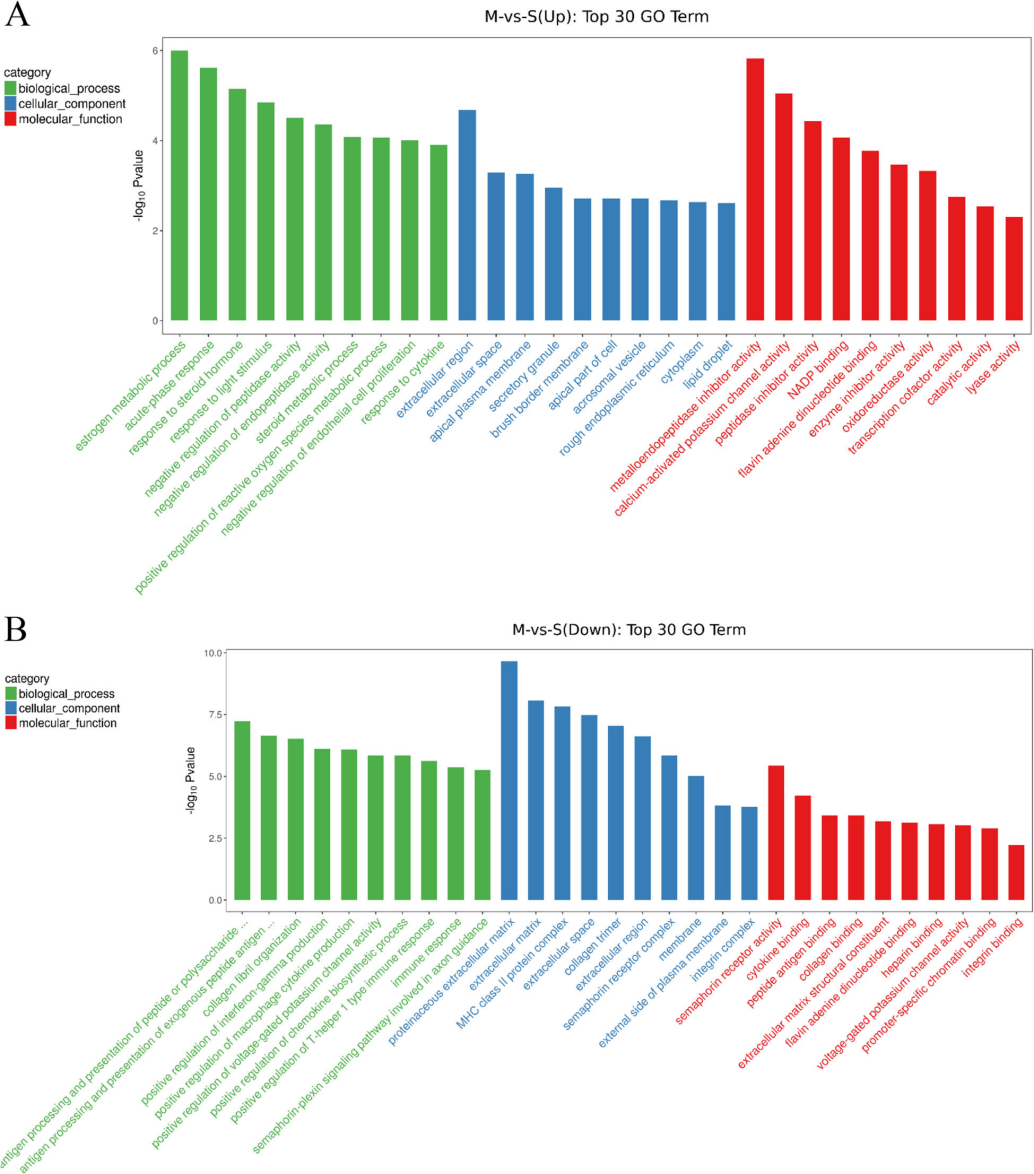
Gene Ontology (GO) enrichment analysis for differentially expressed genes (DEGs). (**A**) Results of GO enrichment analysis for up-regulated DEGs, and (**B**) down-regulated DEGs from middle cerebral artery occlusion (MCAO)-induced mouse heart

The top 30 GO-terms of down-regulated DEGs with the highest enrichment factor are shown in Fig. 4A. Enrichment analysis showed that a total of 319 down-regulated DEGs were significantly enriched in the top 30 GO-terms, 45 of which were in biological processes (BP), 237 of which were in cellular components (CC), and 37 of which were in molecular functions (MF) with *P* < 0.05. The top 5 enriched BP terms were immune response, collagen fibril organization, positive regulation of interferon-gamma production, antigen processing and presentation of exogenous peptide antigen via major histocompatibility complex (MHC) class II, and antigen processing and presentation of peptide or polysaccharide antigen via MHC class II. The top 7 enriched CC terms were membrane, extracellular (region, space, and matrix), proteinaceous extracellular matrix, external side of plasma membrane, and collagen trimer. The top 5 enriched MF terms were heparin binding, collagen binding, flavin adenine dinucleotide binding, voltage-gated potassium channel activity, and integrin binding. These results showed that MCAO suppressed immune response and collagen synthesis, and the DEGs existed in the membrane or extracellular region of cardiomyocytes.

The top 30 GO-terms of up-regulated DEGs with the highest enrichment factor are shown in Fig. 4B. Enrichment analysis showed that a total of 235 up-regulated DEGs were significantly enriched in the top 30 GO-terms, 39 of which were BP, 146 of which were CC, and 50 of which were MF with *P* < 0.05. The top 5 enriched BP terms were negative regulation of peptidase activity, negative regulation of endopeptidase activity, steroid metabolic process, acute-phase response and response to cytokine. The top 5 enriched CC terms were cytoplasm, extracellular region, extracellular space, apical plasma membrane and secretory granule. The top 5 enriched MF terms were oxidoreductase activity, catalytic activity, peptidase inhibitor activity, flavin adenine dinucleotide binding and lyase activity. These results show that modified activity of oxidoreductase, peptidase, and endopeptidase may be involved in MCAO cardiomyopathy. The up-regulation of DEGs mainly existed in the cytoplasm and extracellular region of cardiomyocytes.

### KEGG analysis of DEGs in stroke mouse heart

**Fig. 5:**
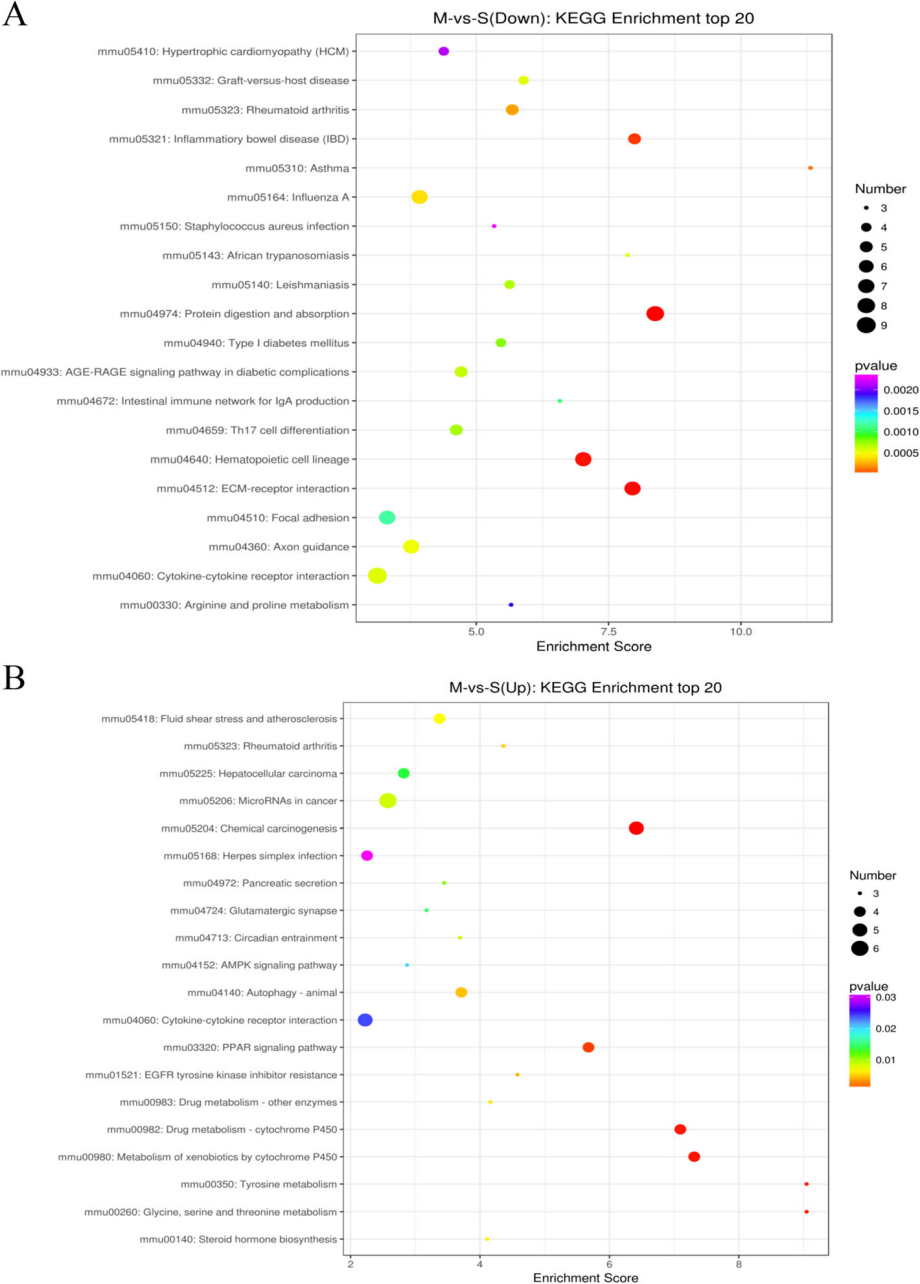
Kyoto Encyclopedia of Genes and Genomes (KEGG) enrichment analysis for differentially expressed genes (DEGs). (**A**) Results of KEGG enrichment analysis for down-regulated DEGs, and (**B**) up-regulated DEGs in middle cerebral artery occlusion (MCAO)-induced mouse heart. Bubble chart of the top 20 significantly enriched pathways for the down-regulated DEGs

Pathway analysis using KEGG can provide further insights into gene functions and their interactions. The top 20 enriched pathways are presented in Fig. 5A and arranged according to the number of down-regulated DEGS. The top 10 pathways of down-regulated DEGs with the greatest enrichment were cytokine-cytokine receptor interaction, protein digestion and absorption, extracellular matrix (ECM)-receptor interaction, hematopoietic cell lineage, influenza A, axon guidance, focal adhesion, inflammatory bowel disease (IBD), rheumatoid arthritis, and advanced glycation end-products/receptor for advanced glycation end-products (AGE-RAGE) signaling pathway in diabetic complications.

The top 20 enriched pathways are presented in Fig. 5B and arranged according to the number of up-regulated DEGS. The top 10 pathways of up-regulated DEGs with the greatest enrichment were microRNAs in cancer, chemical carcinogenesis, cytokine-cytokine receptor interaction, metabolism of xenobiotics by cytochrome P450, drug metabolism-cytochrome P450, PPAR signaling pathway, autophagy-animal, fluid shear stress and atherosclerosis, hepatocellular carcinoma, herpes simplex infection and tyrosine metabolism.

Overall, the analysis of top-ranked pathways showed that genes in the collagen metabolic pathway such as *Col1a1, Col4a6*, and *Col6a3* were suppressed, whereas those in the cytochrome P450 and PPAR signaling pathways were activated, and the cytokine-cytokine receptor interaction pathway was dysregulated in the MCAO mouse heart.

### Verification of DEGs by qPCR

**Fig. 6:**
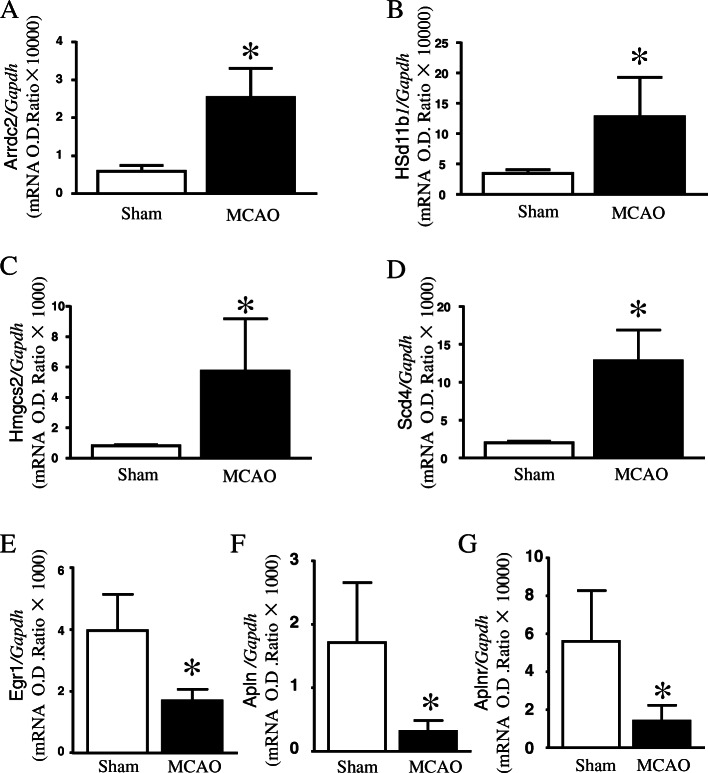
Quantitative assessment of identified differentially expressed genes (DEGs) in mouse heart. Expression of the identified DEGs from RNAseq experiments were quantified using quantitative PCR (qPCR). (**A-D**) mRNA expression of *Arrdc2, Hsd11b1, Hmgcs2,* and *Scd4* in mouse heart. (**E-G**) mRNA expression of *Egr1, Apln,* and *Aplnr* in mouse heart. Data are presented as mean ± SD, *n* = 6. *P < 0.05 vs sham-operated

Based on the FC, GO, and pathway results, we selected 7 genes among all identified DEGs for verification by qPCR. As shown in Fig. 6A-D, *Arrdc2, Hsd11b1, Hmgcs2,* and *Scd4* were significantly up-regulated, whereas *Egr1, Apln,* and *Aplnr* were significantly down-regulated in the MCAO mouse heart (Fig. 6E-G).

### Detection of mRNA of proinflammatory factor

**Fig. 7:**
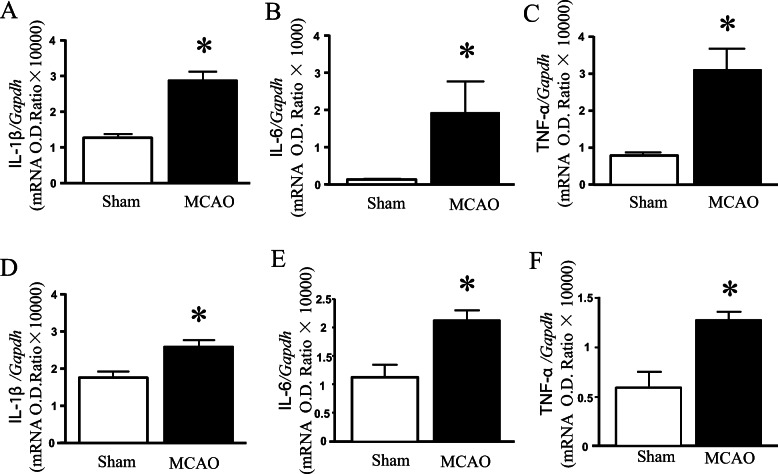
Expressions of pro-inflammatory factor mRNAs between heart and brain in mouse. (**A-C**) The levels of IL1β, IL-6, TNF-α mRNA in the heart. (**D-F**) The levels of IL1β, IL-6, TNF-α mRNA in the brain

In order to further observe the similarity of inflammatory response in heart and brain, we detected the mRNA levels of IL1β, IL-6, TNF-α. Whether in the heart or the brain, the expression of IL1β, IL-6, TNF-α mRNA was up-regulated in MCAO mouse hearts (Fig. 7).

## Discussion

Despite the existence of cardioembolic stroke [26], there is sufficient evidence in many clinical and animal investigations indicating that stroke could induce cardiac injury [5, 10, 27]. However, little is known about the mechanism of stroke-induced cardiomyopathy. This study revealed three major new findings: (1) experimental induced stroke caused cardiac dysfunction at 4 d; (2) atrophic phenotype of the heart occurred after induced stroke; and (3) a unique myocardial mRNA expression profile was produced after induced stroke.

### Stroke induced progressive cardiac dysfunction in adult mice

Cardiac dysfunction is frequently encountered in various forms of stroke such as ischemic stroke [10], brain hemorrhage [28] and subarachnoid hemorrhage [29]. Therefore, neural deregulation and systemic inflammation are considered to be the common mechanism of brain-heart interaction [11, 30]. Based on the mechanism of neuro-regulation mediated cardiac function, cardiac dysfunction occurs in the acute phase. Approximately 67% of acute ischemic stroke patients have ischemic and/or arrhythmic electrocardiogram abnormalities in the first 24 h after a stroke [31]. During the first 3 months after acute ischemic stroke, 19.0% of patients suffer from at least one serious cardiac adverse event; 28.5% have LVEF of less than 50%; and 13–29% have systolic dysfunction [32]. In animal experiments, 28 days after stroke, significantly decreased cardiac function was identified by decreased LVEF compared to non-stroke mice [7]. However, other studies suggest that left ventricular contractility was reduced earlier (24–72 h) but not later (2 months) after brain ischemia in mice subjected to filament-induced left MCAO [12].

Impaired cardiac function as a consequence of severe acute ischemic stroke is a predictor of worse functional outcome and also contributes substantially to the long-term prognosis of ischemic stroke patients [33]. Therefore, investigating the early stage of brain-heart interaction after stroke is highly clinically significant. From other longitudinal studies, cardiac function was maximally decreased within 2–4 days after MCAO [12]. And although we selected the appropriate monofilament, the location and extent of neurological deficit and infarction in MCAO mice in the same group may not be consistent due to the difference of vascular diameter. From the side, a certain degree of cerebral infarction will produce adverse effects on cardiac function and injury.

In our experimental study, we found a substantial reduction of EF%, FS%, and E/A ratio in the early and subacute phase after stroke in previously healthy mice with intact cardiac function, with the decreased total blood flow and the decreased coronary reserve capacity. Our results suggest that close monitoring for cardiac dysfunction is necessary during the early stage. However, further study is needed to determine if cardiac therapy during the early stage can accelerate cerebral ischemia recovery.

### Stroke leads to cardiac atrophy and myocardial remodeling

Cardiac dysfunction relates to the morphological phenotype change in mice after stroke. Previous studies reported cardiac hypertrophy as a long-term (8 week) cardiac consequence of a focal cerebral ischemia [9]. Furthermore, myocyte cross-sectional area was significantly increased compared to non-stroke mice 28 days after stroke. In contrast, mice in our study (MCAO, 60 min ischemia) showed that heart weight rapidly decreased and cardiomyocyte size reduced at 4 days. This was consistent with other researcher who also detected a reduced heart weight/tibia length ratio and cardiomyocyte cross-sectional diameter, which normalized after 14 days [12]. Cardiac phenotype observed in stroke-induced mouse heart did not result in myocardial hypertrophy, but this might have changed over time with deterioration of heart function.

Additionally, expression of collagens that are normally involved in pathological cardiac remodeling was not increased (*collagen-1* and *collagen-3*) in our induced-stroke mouse heart. Furthermore, GO and pathway analyses indicated that stroke induced collagen synthesis related DEGs were down-regulated. A recent study which was consistent with our results, reported that the expression of *col3a1* was reduced, and that of the E3 ubiquitin ligase atrogin-1 and muscle ring finger protein MuRF-1, which are known to regulate skeletal muscle and cardiac atrophy, was increased in stroke-induced mouse heart [12]. Previously, tail-suspension for 28 days, which simulated spaceflight or microgravity conditions, decreased cardiomyocyte size and heart weight, increased MuRF-1 protein levels, and cause myocardial atrophy and dysfunction [34]. Recent animal study data indicate that anthracycline-caused cardiac atrophy is dependent on MuRF-1 [35]. The changes detected at the early stage of stroke showed remodeling characteristics typical of myocardial atrophy as observed due to weightlessness and cancer [36]. MuRF-1 and collagen may be very important mediating mechanisms. However, whether the treatment of short-term myocardial atrophy can prevent long-term myocardial fibrosis and hypertrophy, deserves further investigation.

### Stroke altered transcriptome characteristics

Acute ischemic and hemorrhagic strokes in some particular areas of the brain can disrupt central autonomic control of the heart, precipitating cardiac arrhythmias, myocardial injury and sometimes sudden death [37]. Therapeutically, beta-blockade with metoprolol inhibits sympathetic activity, which then prevents the development of chronic cardiac dysfunction by decelerating cardiac remodeling in ischemic stroke mice [9]. Further study is needed on the heart effects caused by stroke, neural regulation, and cardiomyocyte changes produced at the cell and biological levels.

Stroke induced cardiomyopathy has a unique transcriptome expression profile, different from myocardial infarction [38] and cardiac hypertrophy [39]. Veltkamp [12] reported that several DEGs were up-regulated in the heart 24 h after stroke. However, we found that there were more down-regulated DEGs in MCAO-induced mouse heart. The most significant feature was that stroke-induced hearts had an inhibited immune response. Further, DEGs of cytokine-cytokine receptor interaction, including *Il11, Il18rap, Il22ra1,* and *Il6ra,* were moderately increased. These findings support recent reports that splenectomy with stroke significantly reduces macrophage infiltration into the heart, decreases inflammatory factor expression in the heart, and significantly improves cardiac function compared to non-splenectomized adult stroke mice [11]. Further, *Cxcr4* deficiency reduces innate-immune-system-mediated defense response, which is associated with a deteriorating outcome after transient cerebral ischemia [40].

Unexpectedly, unlike the common pathological remodeling, many collagen metabolic genes were decreased in the stroke-induced heart. These DEGs are mainly distributed in the extracellular matrix, extracellular region, and collagen trimer. Correspondingly, negative regulation of peptidase and endopeptidase was increased in MCAO mouse heart, which may further reduce collagen content. Similarly, autophagy, a protein degradation pathway, was shown to be involved in cardiac dysfunction regulation and tail-suspension induced atrophy. Therefore, disruption of the collagen pathway may play an important role in the cardiac atrophy phenotype. Meanwhile, we found that proinflammatory factors increased in both brain and heart, which is consistent with our sequencing results. The brain-heart interactions pathways disrupted by increased pro-inflammatory responses and reduced immunocompet- ence may be one of the important mechanisms of stroke-induced cardiomyopathy.

In addition, DEGs for oxidoreductase and catalytic activities in the cytoplasm were increased. Of these, we verified that *Hsd11b1* and *Hmgcs2* were consistent with the sequencing results. These results suggested that activated protein degradation pathway and inhibited collagen reduction caused by stroke contribute to myocardial atrophy. Importantly, we also confirmed that the expression of *Apln* and *Aplnr* significantly decreased after stroke.

The expression of apelin was reduced in an age-dependent manner in humans and rodents and was positively associated with the beneficial effects of exercise in older adults [41]. Tail-suspension for 28 days decreased cardiomyocyte size and heart weight, and reduced expression of *Apln* and *Aplnr* [34]. It was also reported that endogenous apelin plays a crucial role in suppressing angiotensin II-induced cardiac dysfunction and pathological remodeling [42]. However, apelin as the target of an innovative pharmacological strategy to prevent stroke-associated myocardial atrophy needs further research.

In conclusion, the experimental induced stroke caused cardiac dysfunction has been well proved and atrophic phenotype of the heart may be an important factor leading to stroke induced cardiomyopathy. Combined with a unique myocardial mRNA sequencing, we screened the differentially expressed miRNA genes that may lead to myocardial atrophy or myocardial dysfunction. To explore the exact relationship between stroke and cardiomyopathy and the interactions between various mediators is the focus of our next study.

## Supplementary Information

**Additional file 1 Fig. S1**

**Additional file 2 Fig. S2**

**Additional file 3 Fig. S3**

**Additional file 4 Table S1**

**Additional file 5 Table S2**

## Data Availability

All supporting data can be found within the manuscript and its supplementary files. The datasets analysed during the current study are available in the NCBI under the accession number PRJNA731991 [https://www.ncbi.nlm.nih.gov/sra/PRJNA731991]. And the corresponding author Dr. Li can be contacted to access the data by email (lishengcun@wmu.edu.cn).
