# Supplementary material for: Ischemic stroke induces cardiac dysfunction and alters transcriptome profile in mice
Source: BMC Genomics. 2021 Sep 4;22:641. doi: 10.1186/s12864-021-07938-y (PMC8418010; doi:10.1186/s12864-021-07938-y)
Supplement: Supplementary file 1 — Additional file 1 Fig. S1 [file 12864_2021_7938_MOESM1_ESM.pdf]

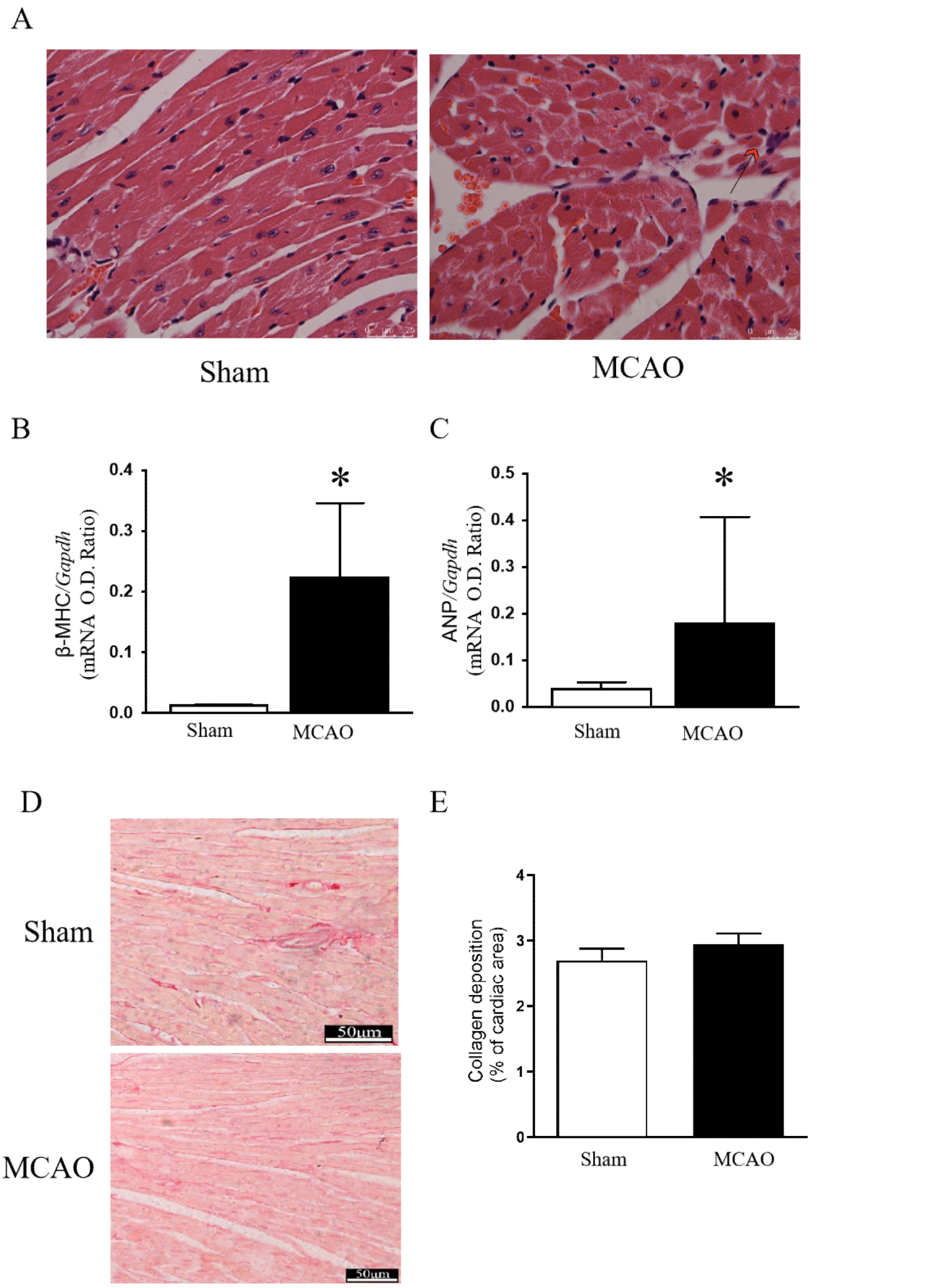

Supplemental Figure S1. Myocardial remodeling in the heart after stroke. (A) Hematoxylin and eosin (HE) staining in the heart tissue at 4 days after stroke. (B, C) The mRNA levels of *□-MHC* and *ANP* genes normalized to the level of Gapdh. (D,E) Representative and quantitation of picro-sirius red staining for collagen deposition (red). Data are presented as mean ± SD, n = 6. *P < 0.05 vs sham-operated.
