# Supplementary material for: Ischemic stroke induces cardiac dysfunction and alters transcriptome profile in mice
Source: BMC Genomics. 2021 Sep 4;22:641. doi: 10.1186/s12864-021-07938-y (PMC8418010; doi:10.1186/s12864-021-07938-y)
Supplement: Supplementary file 2 — Additional file 2 Fig. S2 [file 12864_2021_7938_MOESM2_ESM.pdf]

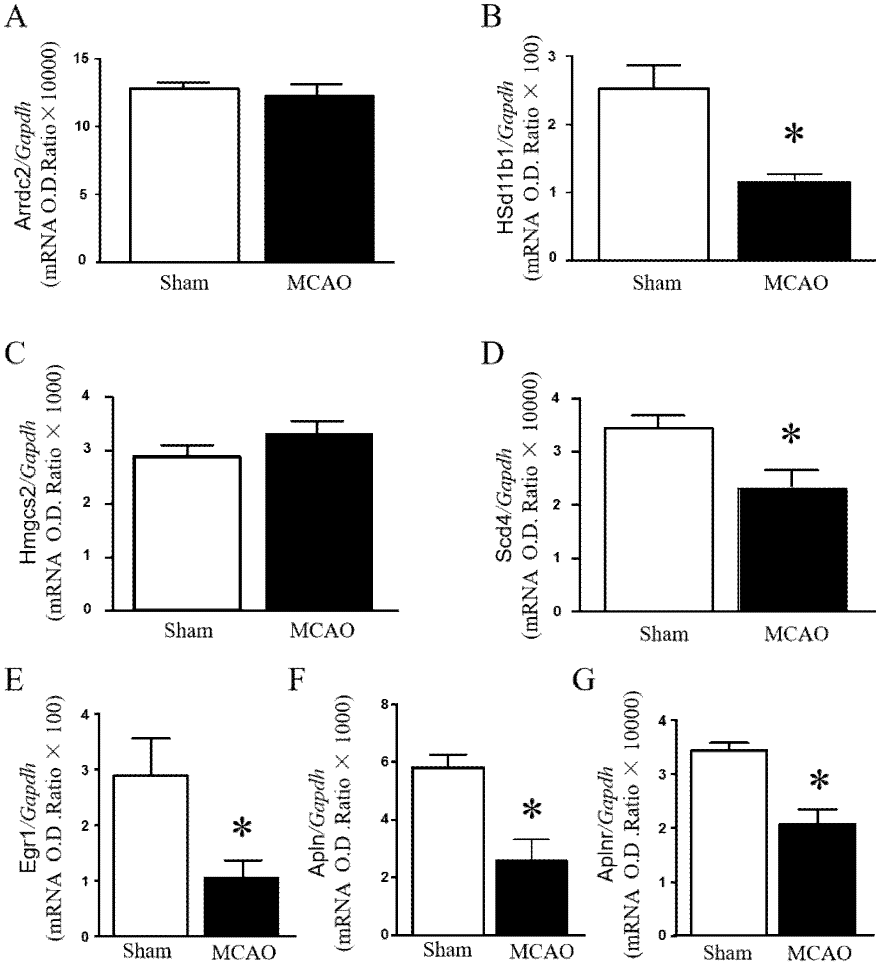

Supplemental Figure S2. The mRNA expession levels of genes in heart. Data are presented as mean ± SD, n = 6. *P < 0.05 vs sham-operated.
