# Supplementary material for: Ischemic stroke induces cardiac dysfunction and alters transcriptome profile in mice
Source: BMC Genomics. 2021 Sep 4;22:641. doi: 10.1186/s12864-021-07938-y (PMC8418010; doi:10.1186/s12864-021-07938-y)

Atrogin-1 (42kDa): s=Sham, m=MCAO Model

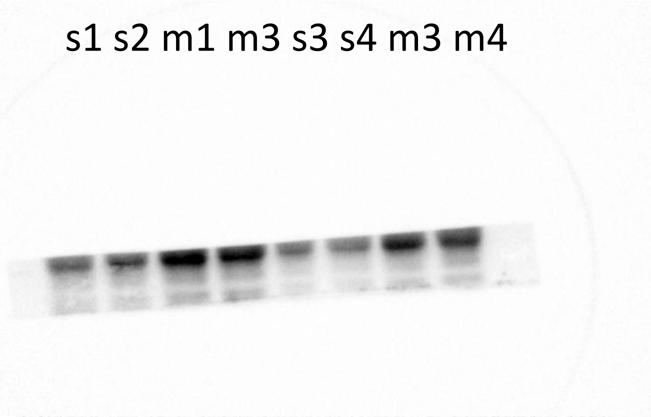

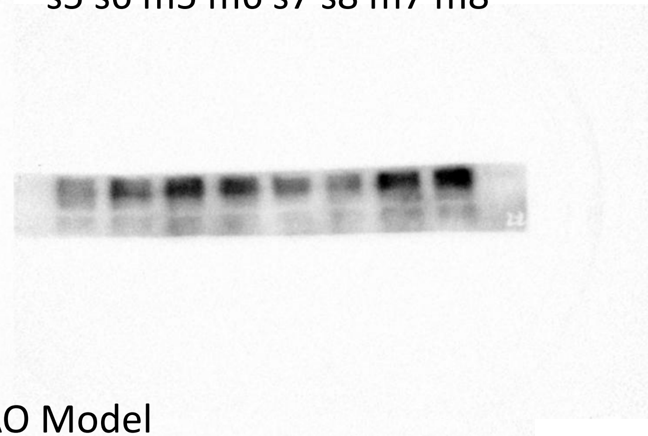

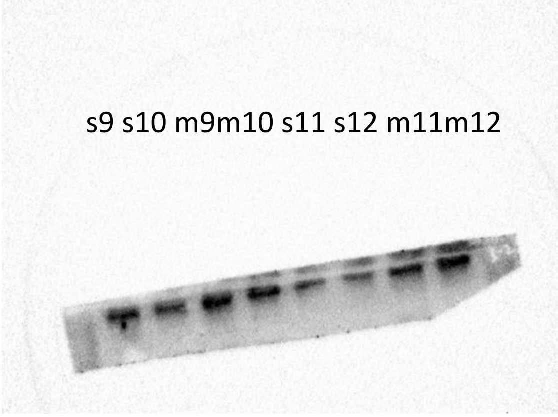

GAPDH (37 kDa): s=Sham, m=MCAO Model

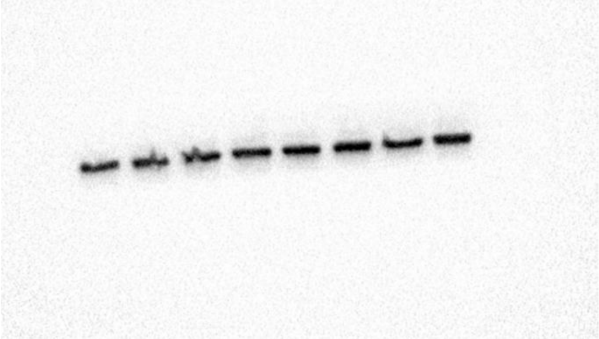

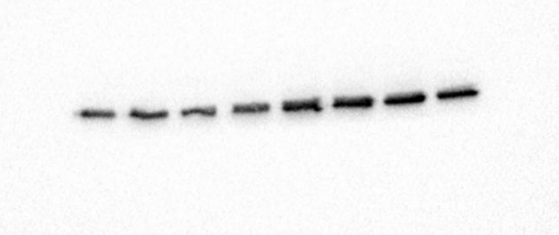

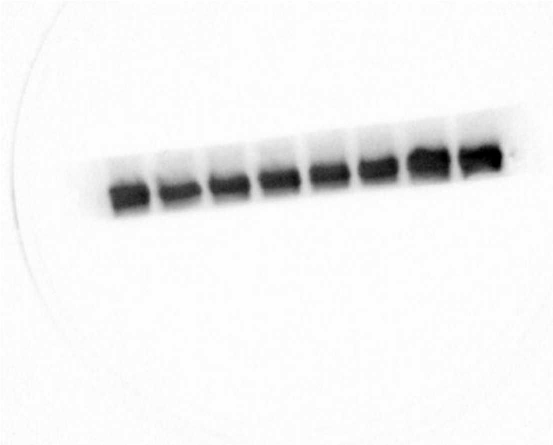

MuRF-1(40kDa): s=Sham, m=MCAO Model

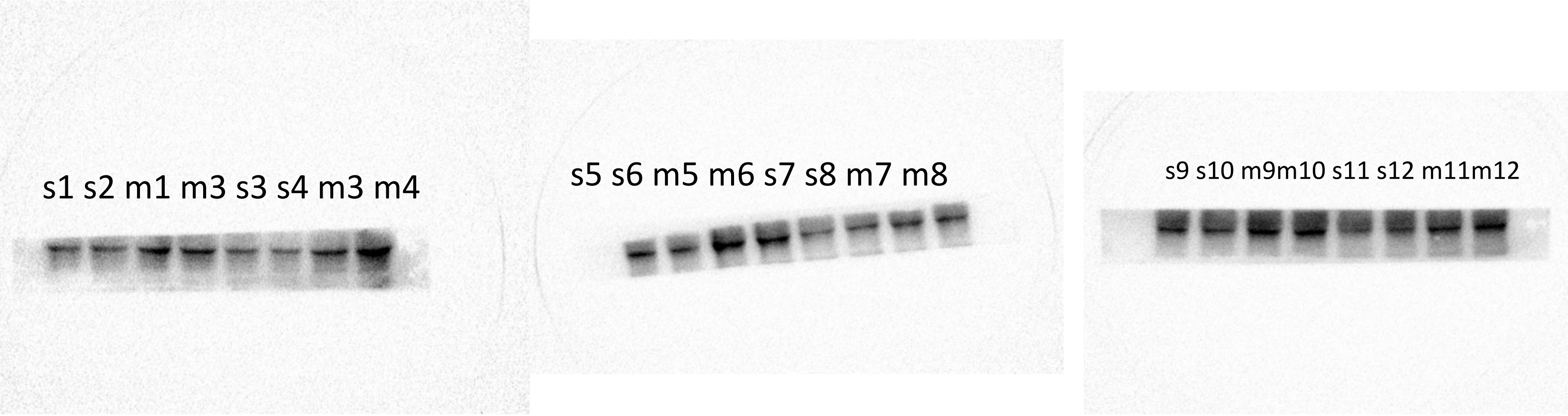

GAPDH (37 kDa): s=Sham, m=MCAO Model

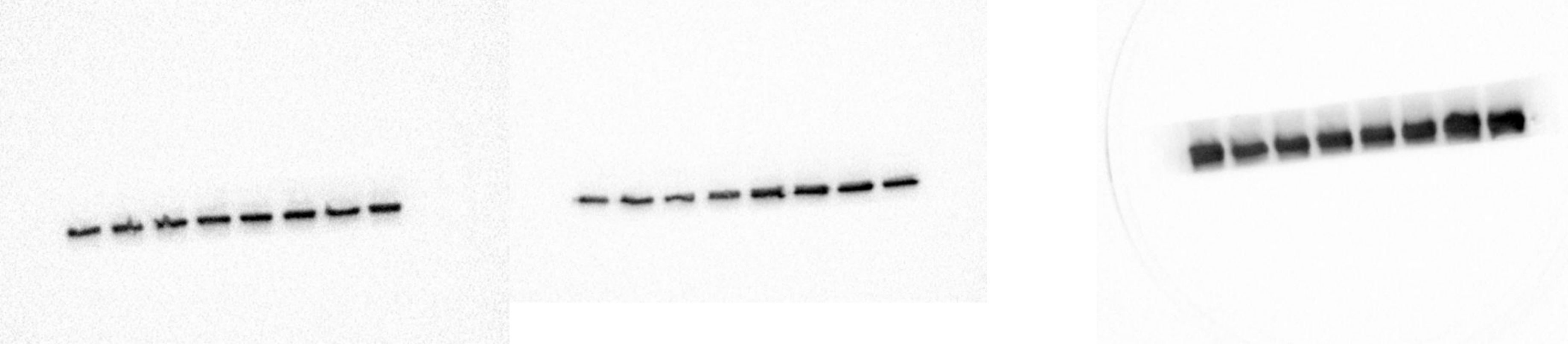

Supplement: Supplementary file 3 — Additional file 3 Fig. S3 [file 12864_2021_7938_MOESM3_ESM.pdf]
