# Supplementary material for: Ischemic stroke induces cardiac dysfunction and alters transcriptome profile in mice
Source: BMC Genomics. 2021 Sep 4;22:641. doi: 10.1186/s12864-021-07938-y (PMC8418010; doi:10.1186/s12864-021-07938-y)
Supplement: Supplementary file 4 — Additional file 4 Table S1 [file 12864_2021_7938_MOESM4_ESM.pdf]

Supplemental Table S1. Primers for qPCR

Target gene	Forward (5′→ 3′)	Reverse (5′ →3′)
ANP	CTGCTAGACCACCTGGAGGA	AAGCTGTTGCAGCCTAGTCC
β-MHC	TGCAAAGGCTCCAGGTCTGAGGGC	GCCAACACCAACCTGTCCAAGTTC
Collagen-1	ACGGCTGCACGAGTCACAC	GGCAGGCGGGAGGTCTT
Collagen-3	GTTCTAGAGGATGGCTGTACTAAACACA	TTGCCTTGCGTGTTTGATATTC
atrogin-1	AGTGAGGACCGGCTACTGTG	GATCAAACGCTTGCGAATCT
MuRF-1	AGAGTGAGCTGAGCGATGG	GTCTGCGGCTGTTGTCCT
Arrdc2	GTGGAAGTCGTGAACCACCG	GCTGGAAGCTGAAGGGAAACT
Hsd11b1	GGAGCCCATGTGGTATTGACT	CCGCAAATGTCATGTCTTCCAT
Hmgcs2	ACCGTGCTCCCTTCTTAGGT	GCTCTTCGTGGGTTCTGTGT
Scd4	GTACTTGTGGGGTGAGACTTTTC	CACCAATGCCCCTGTCATAG
Egr1	AGACGAGTTATCCCAGCCAAA	GGTCGGAGGATTGGTCATGC
Apln	CCA CTG ATG TTG CCT CCA GA	GCG AAA TTT CCT CCT GCC TC
Aplnr	GCTCATTCCTGCCATCTACA	AAAGTCACCACAAAGGTCAAGT
Gapdh	CAGTGTTGGGGGCTGAGTTG	AAAGGGCATCCTGGGCTACA
IL-1β	GAAGAAGTGCCCATCCTCTG	AGCTCATATGGGTCCGACAG
TNF-α	CCGATGGGTTGAACCTTGTC	GGGCTGGGTAGAGAATGGAT
IL-6	TCACAGAAGGAGTGGCTAAGGACC	ACGCACTAGGTTTGCCGAGTAGAT
