# Supplementary material for: Ischemic stroke induces cardiac dysfunction and alters transcriptome profile in mice
Source: BMC Genomics. 2021 Sep 4;22:641. doi: 10.1186/s12864-021-07938-y (PMC8418010; doi:10.1186/s12864-021-07938-y)
Supplement: Supplementary file 5 — Additional file 5 Table S2 [file 12864_2021_7938_MOESM5_ESM.pdf]

Supplemental Table S2. Heart rate in the initial state of anesthesia.

Mice	Systolic	Diastolic
Sham01	525	413
Sham02	518	499
Sham03	415	428
Sham04	468	465
average	481.5	451.25
MCAO01	312	288
MCAO02	325	296
MCAO03	444	377
MCAO04	276	276
average	339.25	309.25

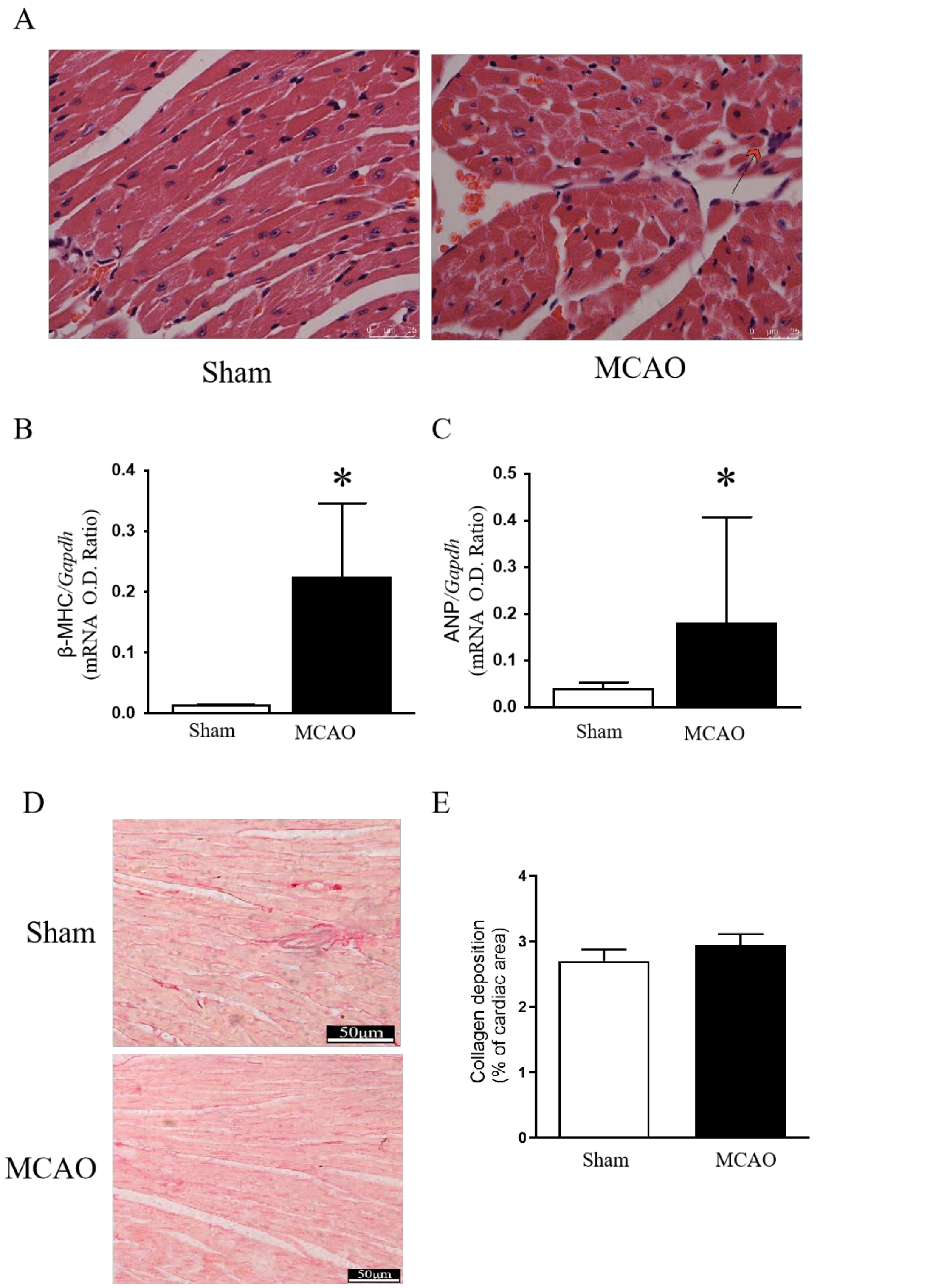

Supplemental Figure S1. Myocardial remodeling in the heart after stroke. (A) Hematoxylin and eosin (HE) staining in the heart tissue at 4 days after stroke. (B, C) The mRNA levels of *β-MHC* and *ANP* genes normalized to the level of Gapdh. (D,E) Representative and quantitation of picro-sirius red staining for collagen deposition (red). Data are presented as mean ± SD, n = 6. *P < 0.05 vs sham-operated.

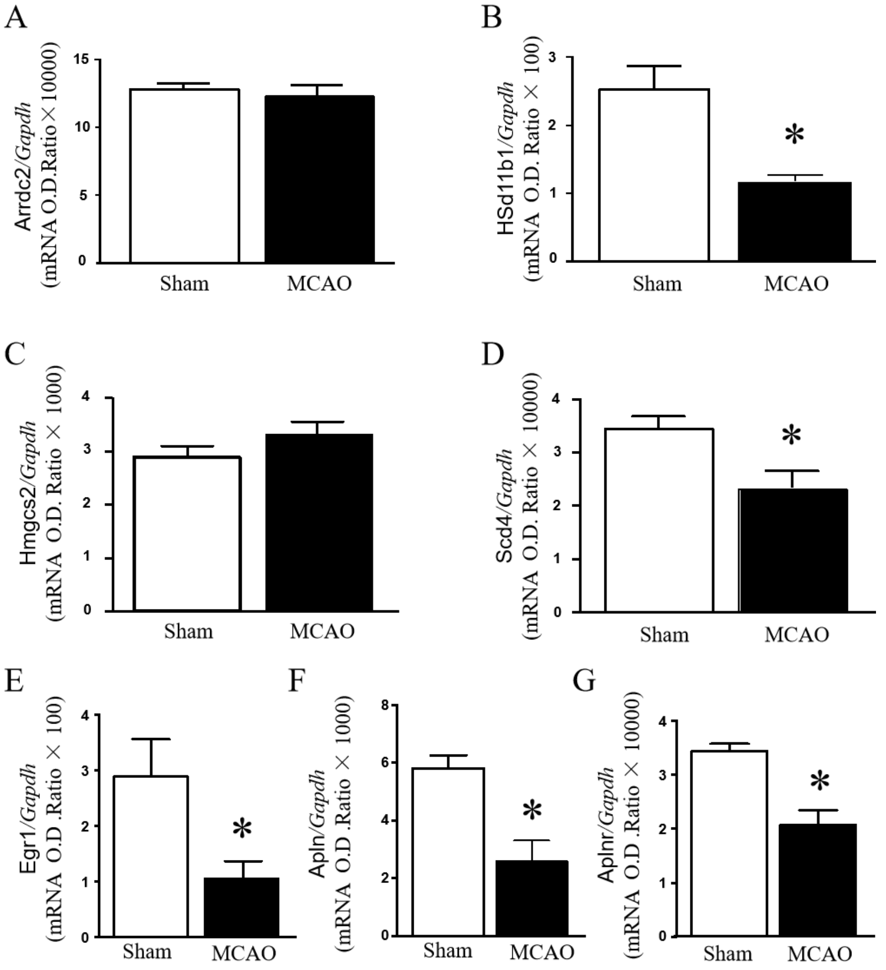

Supplemental Figure S2. The mRNA expession levels of genes in heart.
